# Unveiling the Antioxidant, Clinical Enzyme Inhibitory Properties and Cytotoxic Potential of *Tambourissa peltata* Baker—An Understudied Endemic Plant

**DOI:** 10.3390/molecules28020599

**Published:** 2023-01-06

**Authors:** Shanoo Suroowan, Eulogio J. Llorent-Martínez, Gokhan Zengin, Kalaivani Buskaran, Sharida Fakurazi, Ashraf N. Abdalla, Asaad Khalid, Bao Le Van, Mohamad Fawzi Mahomoodally

**Affiliations:** 1 Department of Health Sciences, Faculty of Medicine and Health Sciences, University of Mauritius, Réduit 80837, Mauritius; 2 Department of Physical and Analytical Chemistry, University of Jaén, Campus Las Lagunillas S/N, 23071 Jaén, Spain; 3 Department of Biology, Science Faculty, Selcuk University, Konya 42130, Turkey; 4 Laboratory of Natural Medicine and Product Research, Institute of Bioscience Universiti Putra Malaysia, Serdang 43400, Malaysia; 5 Department of Human Anatomy, Faculty of Medicine & Health Sciences, Universiti Putra Malaysia, Serdang 43400, Malaysia; 6 Department of Pharmacology and Toxicology, College of Pharmacy, Umm Al-Qura University, Makkah 21955, Saudi Arabia; 7 Substance Abuse and Toxicology Research Center, Jazan University, P.O. Box 114, Jazan 45142, Saudi Arabia; 8 Medicinal and Aromatic Plants and Traditional Medicine Research Institute, National Center for Research, Khartoum P.O. Box 2404, Sudan; 9 Institute of Research and Development, Duy Tan University, Da Nang 550000, Vietnam; 10 Faculty of Natural Sciences, Duy Tan University, Da Nang 550000, Vietnam; 11 Center for Transdisciplinary Research, Department of Pharmacology, Saveetha Institute of Medical and Technical Science, Saveetha Dental College, Chennai 600077, India; 12 Centre of Excellence for Pharmaceutical Sciences, North-West University, Private Bag X6001, Potchefstroom 2520, South Africa

**Keywords:** *Tambourissa peltata*, antioxidant, phenolic, cytotoxicity

## Abstract

This study documents for the first time the phytochemical composition and biological activities of *Tambourissa peltata* Baker, an endemic plant from Mauritius. Phytochemical extraction was performed using ethyl acetate, methanol and distilled water as solvents. The phytochemical composition was determined through HPLC-MS and other standard assays. The DPPH, ABTS, FRAP, CUPRAC and phosphomolybdenum assays were employed for the determination of the antioxidant potential, whereas cell viability assays were used to determine the cytotoxicity. The highest phenolic and phenolic acid contents were obtained in the aqueous extract (179.91 ± 0.67 gallic acid equivalents/g and 55.74 ± 1.43 caffeic acid equivalents/g). The highest quantity of flavonoids was obtained in the ethyl acetate extract (28.97 ± 0.46 rutin equivalents/g). The methanolic extract was the highest source of flavonols (33.71 ± 0.13 mg catechin equivalents/g). A total of 34 phytochemicals were identified, mainly proanthocyanidins and flavonoid glycosides. The highest antioxidant activity in DPPH (973.40 ± 5.65 mg TE (Trolox equivalents)/g), ABTS (2030.37 ± 40.83 mg TE/g), FRAP (1461.39 ± 5.95 mg TE/g), CUPRAC (1940.99 ± 20.95 mg TE/g) and phosphomolybdenum (8.37 ± 0.23 mmol TE/g) assays was recorded for the aqueous extract. The ethyl acetate extract was the most active metal chelator. The highest acetylcholinesterase inhibitor was the methanolic extract, whereas the ethyl acetate extract was the most active against BChE. The tyrosinase enzyme was most inhibited by the methanolic extract. Alpha-amylase and glucosidase were most inhibited by the aqueous extract. The methanolic extract was capable of inducing cell cytotoxicity to the human colorectal carcinoma without damaging normal cells. *T. peltata* warrants further attention from the scientific community given its multifaceted biological properties.

## 1. Introduction

Mauritius is a small island lying in the southern hemisphere of the Indian Ocean bearing coordinates 57°30′ east and 20°20′ south. The island withholds a dense flora with a reported number of over 711 flowering plant species, among which 246 are endemic [1]. Despite its richness in endemic plants, most of them have not been evaluated with regard to their pharmacological properties.

One such endemic plant *T. peltata*, belonging to the Monimiaceae family, has been evaluated regarding its antibacterial and antifungal properties against *Escherichia coli* (ATCC 25922), *Staphylococcus aureus* (ATCC 29213), *Enterococcus faecalis* (ATCC 21212), *Pseudomonas aeruginosa* (ATCC 27853), *Aspergillus niger* (ATCC 16404) and *Candida albicans* (ATCC 26219) only, leaving its phytochemical composition and other pharmacological properties unexplored [2]. Species from the Monimiaceae plant family are renowned for their uses in traditional medicine systems and known to be good sources of secondary metabolites bearing noticeable biological and therapeutic properties, mostly recognized for potential antioxidant and anticancer propensities [3].

Interestingly, one of the most researched qualities of plant species relates to their antioxidant potential. Compounds with antioxidant potential have been reported as beneficial in the management of severe non-communicable disorders, such as Alzheimer’s disease, atherosclerosis, cerebral and cardiovascular events, diabetes mellitus and hypertension [4].

Alzheimer’s disease causes around 60–70% of cases of dementia, a condition where there is deterioration of cognitive functions, and this condition is a major societal burden for carers, families and society in general [5]. In 2014, 422 million people were living with diabetes mellitus worldwide, and the mortality from the disease rose by 3% between the years 2000 and 2019 [6].

The demand for beauty and personal care products has become more important than ever. By 2025, it is expected that the market for these skin care products will reach an estimated USD 716 billion [7]. Maintaining good care of the skin also ensures remarkable esthetic attributes in modern society [8]. Plant extracts, through their antioxidant effects, can protect the skin from exogenous and endogenous factors [9].

Similarly, the abundance of free radicals in the human body leads to cell aging. The generation of reactive oxygen species can in addition damage DNA and chromosomes, leading to more serious ailments such as cancer [10]. In this event, antioxidants from plant sources are considered healthier and greener than synthetic antioxidants. The importance of adjunctive plant-based antioxidant foods which can neutralize free radicals is consequently key to attenuating deleterious health effects [11].

Targeting enzymes is another approach to drug discovery and pharmacotherapy. The two main targets in the pathogenesis of neurodegenerative disorders (NDs) such as Alzheimer’s and Parkinson’s diseases are acetylcholinesterase and butyrylcholinesterase [12]. They are involved in the hydrolysis of acetylcholine (AChE), which leads to a cholinergic deficit, and this is a common occurrence in most NDs [13]. Inhibition of AChE and BChE can reverse cholinergic deficit to a certain extent, thereby improving ND symptoms [14].

Likewise, inhibition of α-amylase and glucosidase enzymes inhibits carbohydrate digestion and reduces the rate at which glucose reaches the bloodstream. Sugar in the form of simple sugars enters the blood stream following the catalysis of polysaccharides undertaken by α-amylase and glucosidase enzymes. α-Amylase is secreted both by the salivary glands and the pancreas and hydrolyses oligosaccharides such as starch to simple sugars. On the other hand, α-glucosidase is secreted solely by the pancreas and hydrolyzes polysaccharides [15].

The tyrosinase enzyme is involved in melanin biosynthesis. Melanin, being a highly durable substance, is difficult to degrade. Skin lightening therapies focus on firstly inhibiting tyrosinase activity and secondly preventing its biosynthesis by blocking the activation of melanocytes by UVA [16].

On the other hand, in vitro cytotoxicity and cell viability studies are important tools for evaluating compound toxicity and tumor cell inhibition during drug development. For the purpose of this study, they enable us to understand which extracts are toxic to cancer cell lines while at the same time can be harmless to normal cells. For fighting hepatocellular carcinoma and colon carcinoma, the HepG2 and HT29 cell lines have been studied. Given their low operating cost, rapidity and given the fact that no animal subjects are required during this type of screening, this remains a valuable approach for scientists, especially when dealing with a large number of samples in cancer research [17].

Hence, we focused on unveiling the phytochemical and antioxidant potential of *T. peltata* for the first time. We further investigated the biological properties of the plant species through its enzyme inhibitory properties against α-amylase and glucosidase enzymes, acetylcholinesterase and butyrylcholinesterase and tyrosinase enzymes. The goal of this study was to unveil how *T. peltata* can be involved in the development of novel plant-based medicinal extracts and pharmacophores in an attempt to address various ailment conditions that modern science is still struggling to manage. It is anticipated that this preliminary evaluation will establish baseline data that will open new avenues for research on this endemic plant.

## 2. Results

Data for total phenolics, flavonoids, phenolic acids and flavonols content are given in Table 1. The aqueous extract was the highest in total phenolic and total phenolic acid composition, with recorded values of 179.91 ± 0.67 gallic acid equivalents/gram (GAE/g) and 55.74 ± 1.43 caffeic acid equivalents/gram (CAE/g), respectively, followed by the methanolic and ethyl acetate extracts (Table 1). No phenolic acid was found in the ethyl acetate extract, whereas the ethyl acetate extract was highest in flavonoid content (28.97 ± 0.46 rutin equivalents/gram (RE/g)) followed by the methanolic and aqueous extracts. On the other hand, the methanolic extract was the highest source of flavonol with a measured 33.71 ± 0.13 mg catechin equivalents/gram (CE/g) of flavonol, followed by the ethyl acetate and aqueous extracts.

**Figure 1:**
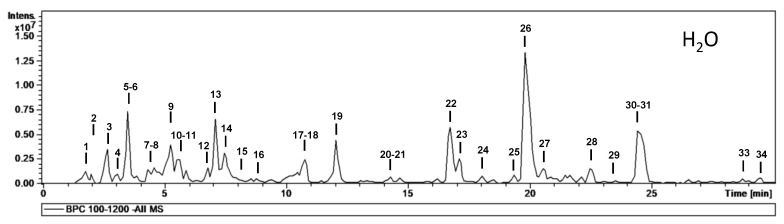
HPLC-ESI-MS^n^ base peak chromatograms of the aqueous extract.

**Figure 2:**
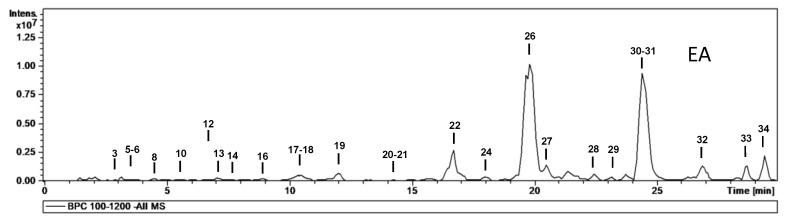
HPLC-ESI-MS^n^ base peak chromatograms of the ethyl acetate extract.

**Figure 3:**
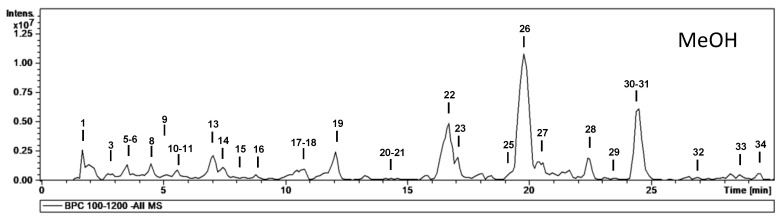
HPLC-ESI-MS^n^ base peak chromatograms of the methanolic extract.

MS data for the characterization of phytochemicals in the different extracts (aqueous, methanolic and ethyl acetate) are given in Table 2, and base peak chromatograms of the aqueous, ethyl acetate and methanolic extracts are shown in Figure 1, Figure 2 and Figure 3, respectively. Most of the compounds identified were proanthocyanidins and flavonoid glycosides. The proanthocyanidins identified can be divided into procyanidins, which consist exclusively of catechin and epicatechin units, and prodelphinidins, which contain (epi)afzelechin or (epi)gallocatechin units. The following is a brief description of the characterization of all compounds.

Compound **1** was characterized as a disaccharide of two hexoses (probably glucose) due to the mass fragmentation pattern [18]. Compounds **2**, **16**, **19** and **32** were identified by comparison with analytical standards. Compound **9** was tentatively characterized as a dihydroxybenzoic acid derivative based on the 153→109 fragmentation (typical of dihydroxybenzoic acid). Compounds **8** and **13** were identified as gallocatechin and epigallocatechin according to the bibliography [19]. The characterization of all proanthocyanidins was performed based on bibliographic information [19,20,21,22]. The identification of flavonoid glycosides was performed based on the neutral losses of 132, 146 and 162 Da, which corresponded to neutral losses of pentoside, deoxyhexoside and hexoside moieties, respectively. The aglycones isorhamnetin, kaempferol, myricetin and quercetin were identified by comparison with analytical standards, whereas mearnsetin was characterized by comparison of its fragmentation pattern with bibliographic information [23].

For the quantification of the main compounds (Table 3), calibration curves (0.5–100 mg mL^−1^) were prepared with the following analytical standards: catechin, protocatechuic acid, procyanidin B2, quercetin, kaempferol, myricetin and isorhamnetin. Repeatability (*n* = 9) and intermediate precision (*n* = 9, 3 consecutive days) were lower than 3 and 8%, respectively. Twenty-seven compounds were individually quantified, and total individual phenolic content (TIPC) was defined as the sum of all the quantified compounds. The aqueous extract presented the highest TIPC (due to the highest solubility of proanthocyanidins), followed by ethyl acetate and methanolic extracts. In the aqueous extract, proanthocyanidins and flavonoids represented 43 and 57% of TIPC, respectively. On the other hand, ethyl acetate and methanolic extracts were composed mostly of flavonoids: 94% and 87% of TIPC in EA and MeOH, respectively. Among the proanthocyanidins, dimers were more abundant than trimers. Among flavonoids, the most abundant ones were myricetin glycosides, which represented approximately 50% of all flavonoids in all the analyzed extracts. Specifically, compound **26** (myricetin-*O*-deoxyhexoside) was the most abundant in all extracts, corresponding to 21, 45 and 39% of TIPC in aqueous, EA and MeOH extracts, respectively.

The antioxidant assay shown in Table 4 demonstrated that the aqueous extract was the most potent antioxidant in the DPPH (973.40 ± 5.65 mg TE/g), ABTS (2030.37 ± 40.83 mg TE/g), FRAP (1461.39 ± 5.95 mg TE/g), CUPRAC (1940.99 ± 20.95 mg TE/g) and phosphomolybdenum (8.37 ± 0.23 mmol TE/g) assays.

In all these assays, the methanolic extract of *T. peltata* followed the aqueous extract. The lowest antioxidant potential was recorded for the ethyl acetate extract. In the metal chelating assay, the ethyl acetate extract showed the highest potential with recorded results of 4.88 ± 0.32 versus 4.75 ± 0.70 mg EDTAE/g for the aqueous extract and 2.12 ± 0.26 mg EDTAE/g for the methanolic extract.

The highest acetylcholinesterase inhibitor was the methanolic extract (4.87 ± 0.04 mg GALAE/g) followed by the ethyl acetate (3.98 ± 0.10 mg GALAE/g) and aqueous extracts (3.49 ± 0.06 mg GALAE/g), respectively. Conversely, the BChE inhibition assay results demonstrated that the ethyl acetate extract inhibited the butyrylcholinesterase enzyme to a greater extent than the methanolic extract and aqueous extracts with recorded values of 9.76 ± 0.14, 5.80 ± 0.12 and 2.83 ± 0.42 mg GALAE/g, respectively. The tyrosinase enzyme was most inhibited by the methanolic extract (155.30 ± 0.55 mg KAE/g) versus the ethyl acetate extract and aqueous extracts (132.77 ± 0.90 and 124.27 ± 1.77 mg KAE/g, respectively). The enzymes involved in the pathogenesis of diabetes amylase and glucosidase were most inhibited by the aqueous extract followed by the methanolic and ethyl acetate extracts. The recorded values for the amylase and glucosidase inhibitory potential were 1.37 ± 0.04 and 1.82 ± 0.01 mmol ACAE/g for the aqueous extract, 0.92 ± 0.02 and 1.80 ± 0.01 mmol ACAE/g for the methanolic extract and 0.72 ± 0.07 and 1.70 ± 0.03 mmol ACAE/g for the ethyl acetate extract. The enzyme inhibitory properties of the extracts are presented in Table 5.

**Figure 4:**
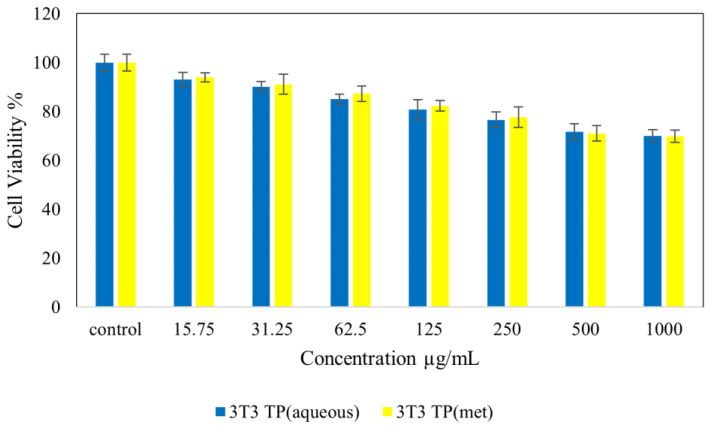
Cytotoxic activity of extracts against normal 3T3 cells at 48 h.

**Figure 5:**
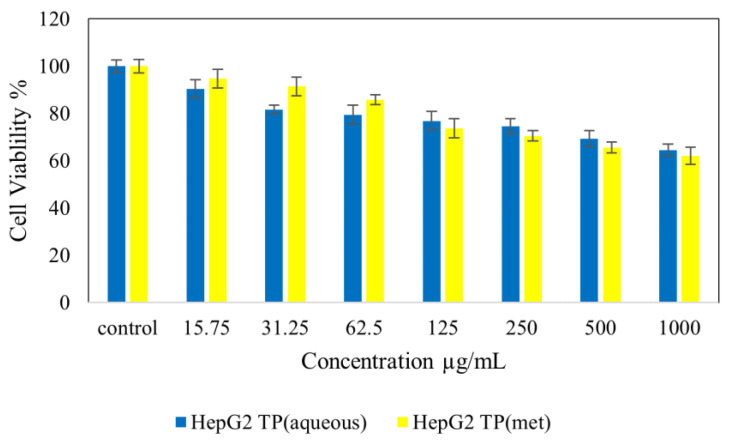
Cytotoxic activity of extracts against normal HepG2 cells at 48 h.

**Figure 6:**
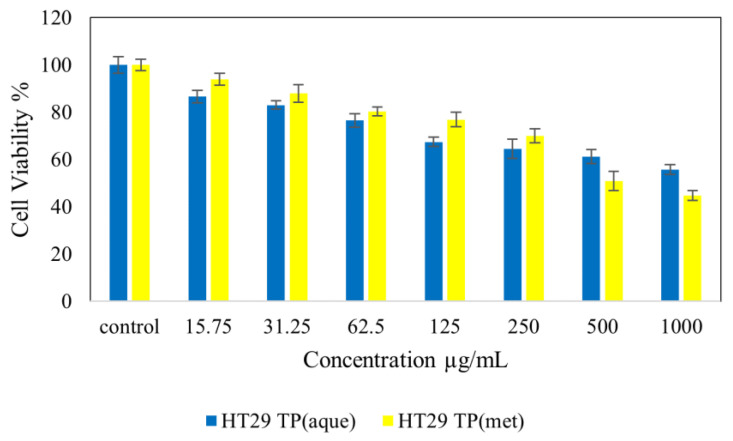
Cytotoxic activity of extracts against HT29 cells at 48 h.

**Figure 7:**
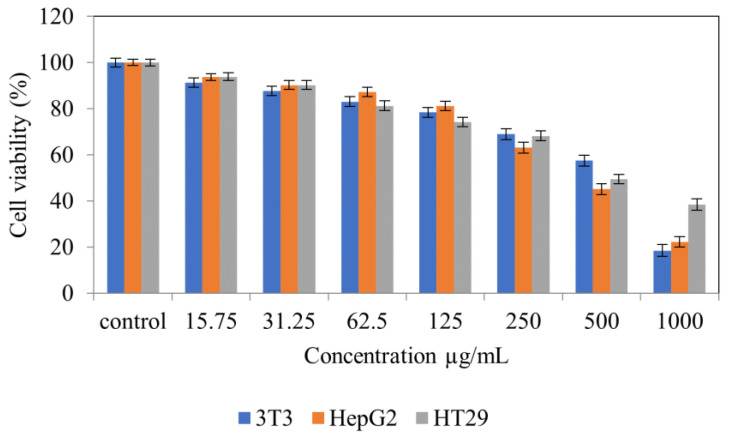
Cytotoxic activity of ethyl acetate extracts against cell lines at 48 h.

Figure 4 shows normal NIH 3T3 cell lines treated with *Tambourissa peltata* Baker. In Figure 5, HepG2 treated with *T. peltata* aqueous and methanolic extracts exhibits nontoxicity up to 250 µg/mL after 48 h of treatment. According to Figure 6, HT29 treated with *Tambourissa peltata Baker* aqueous and methanolic extracts in aqueous started to exhibit nontoxicity up to 125 and up to 250 µg/mL in methanolic extraction. The IC_50_ (µg/mL) value exhibited in methanolic extraction of *T. peltata* at 493.11 µg/mL. The IC_50_ values recorded for the ethyl acetate extract against the 3T3, HepG2 and HT29 cell lines were 722.77, 590.20 and 820.35, respectively (Figure 7). ANOVA revealed that there was no significant difference among the sample groups at individuals from 15.75–250 µg in NIH 3T3 cell concentrations. The significant difference of *p* ≤ 0.05 was exhibited by HepG2 (31.25–500 µg/mL) cells and HT29 (31.25–1000 µg/mL) cells according to the ANOVA and Games–Howell multiple range test in all the cells that were tested. This shows that the methanolic extract of *T. peltata* Baker is capable of inducing cell cytotoxicity in the human colorectal carcinoma without damaging normal cells.

## 3. Discussion

*Tambourissa peltata* Baker is an endemic plant from the island of Mauritius. It is commonly known as ‘bois tambour’ in the Mauritian vernacular language. Up to now, scientific research on this plant shows that it has been investigated for its antibacterial and antifungal properties, especially against *Escherichia coli* (ATCC 25922), *S. aureus* (ATCC 29213), *E. faecalis* (ATCC 21212), *A. niger* (ATCC 16404) and *C. albicans* (ATCC 26219) with documented MIC values of 0.88, 1.14, 1.75, 3.50 and 7 mg/mL respectively [2].

Nonetheless, the phytochemical composition and other biological properties of *T. peltata* have remained unexplored. Hence, this work studied the phytochemical content alongside the biological properties of this species for the first time. *T. peltata* belongs to the Monimiaceae plant family [24]. Plants from this family are usually shrubs or trees bearing essential oils. This plant family is rich in diverse metabolites, which are bioactive, including flavonoids, terpenoids, homogensitic acid derivatives and alkaloids [3].

All extracts contained phenolics, flavonoids, phenolic acids (not present in the ethyl acetate extract) and flavonol. Both phenolic and flavonoid compounds have countless biological activities. Many of these biological activities have medicinal properties. For example, these compounds can be used to promote good health through their anticancer, antioxidant, anti-inflammatory, antibacterial, cardioprotective, immunoprotective as well as skin protective effects among diverse other health benefits [25]. The presence of phenolics and flavonoids in *T. peltata* demonstrates that it is a plant that can be further explored for the potential therapeutic benefits it can induce.

To determine which phytochemicals in an extract have the highest antioxidant potential, liquid chromatography can be employed. For this task, high-performance liquid chromatography is coupled with detectors such as mass spectrometers. This method is routinely employed to identify and isolate individual phytochemical compounds [26]. In this study, a total of 34 phytochemicals were identified from the extracts. Most of the phytochemicals (33) were identified from the aqueous extract, 25 were identified from the ethyl acetate extract while 30 were identified from the methanolic extract.

The main phytochemicals identified from the HPLC analysis were proanthocyanidins and flavonoids. The highest proportion of proanthocyanidins and flavonoids was recorded from the aqueous extract. The methanolic extract was richer in proanthocyanidins than the ethyl acetate extract, while the opposite was noted for the flavonoid content of the extracts.

Proanthocyanidins are also known as condensed tannins, and they are associated with a broad range of biological activities which may exert some therapeutic properties as well. Indeed, the anti-oxidative, anti-inflammatory, anti-obesogenic, anti-cancerous, anti-nociceptive, anti-hyperalgesic, anti-fatigue, anti-fibrotic, anti-virulence, anti-viral and anti-helminthic and hepatoprotective properties of proanthocyanidins are well recognized [27].

Flavonoids such as catechins, epigallocatechins, myricetin, kaempferol and isohamnetin also have notable biological properties. The therapeutic properties that flavonoids can exert are diverse. They exert their antibacterial activities by destroying the bacterial cell wall and decrease inflammation by inhibiting both cytokine and prostaglandin biosynthesis. They can help in diabetes by inhibiting enzymes and glucose transporters, and against Alzheimer’s disease they can delay disease progression by inhibiting cholinesterase enzymes and decreasing the beta amyloid protein. Their anti-cancer potential is also well recognized, and they can exert this property by acting through diverse mechanisms, including (i) inducing apoptosis, (ii) triggering cytotoxicity and (iii) causing cell cycle arrest and scavenging free radicals [28].

The highest antioxidant potential was recorded for the aqueous extract; this can be related to the fact that a greater proportion of phenolic, phenolic acids, flavonoids and proanthocyanidins were found in this extract. A set of seven different antioxidant assays were performed to obtain good validation of the antioxidant properties of the extracts. Interestingly, in the ABTS, FRAP and CUPRAC assays, the aqueous extract exhibited a higher antioxidant potential than the antioxidant used as reference.

Indeed, as per the results of the ABTS assay, the antioxidant potential of the aqueous extract was up to 2-fold higher than Trolox and 1.4- and 1.9-fold higher than the antioxidant potential of Trolox in the FRAP and CUPRAC assays. The methanolic extract demonstrated up to 1.2-fold higher antioxidant potential than the standard Trolox used in the CUPRAC assay. The ethyl acetate extract was the highest metal chelator.

Alzheimer’s disease (AD) has become a major age-related concern globally [29]. Worldwide, it affects around 4.6 million people every year and is one of the most common forms of dementia. The clinical symptoms of the disease are mainly memory impairment, behavioral abnormalities and cognitive dysfunction. Reduced levels of acetylcholine are noted in patients with Alzheimer’s disease.

Alkaloids, flavonoids, terpenoids and phenolics are plant-derived compounds that have shown good potential against AD [30]. Hence, inhibitors of enzymes that are involved in reducing the levels of acetylcholinesterase enzymes in the brain represent an important strategy for finding new AD leads. All three extracts of *T. peltata* showed almost the same activity in acetylcholinesterase enzyme inhibition. While for the inhibition of butyrylcholinesterase, the ethyl acetate extract was the most active inhibitor followed by the methanolic and aqueous extracts.

Tyrosinase is a copper-containing enzyme that plays a role in the production of melanin, which is a type of pigment that can be produced by plants, animals, fungi and bacteria. It is also involved in wound healing, radiation protection and browning of fruits and vegetables [31]. Hyperpigmentation of the skin occurs when there is an overproduction of melanin and causes the skin to blemish and darken. Hence, inhibition of the tyrosinase enzyme is a useful approach to maintaining the appearance of the skin and preventing hyperpigmentation. Synthetic pharmaceutical products such as hydroquinone are available inhibitors of hyperpigmentation but have consequent side effects. Hydroquinone even at a concentration of 1% can exert cytotoxicity on melanocytes. Hence, there is a need for subtle forms of medicine such as plants that maintain the appearance of the skin [32]. All three extracts tested for their anti-tyrosinase activities were active against the enzyme and can be explored, pending further studies, as novel anti-tyrosinase agents.

Alpha-amylase and alpha-glucosidase are the carbohydrate hydrolyzing enzymes found inside the human body that lead to postprandial hyperglycemia. Inhibition of these enzymes can prevent postprandial hyperglycemia in diabetics and prevent diabetes in normal individuals. All extracts were active as inhibitors of the alpha-amylase and alpha-glucosidase. The aqueous extract was most active against the amylase and glucosidase enzymes followed by the methanolic extract and ethyl acetate extracts.

One of the most common malignancies in the world is colorectal cancer; by 2030, its malignancy incidence is expected to grow by 60%. Due to the emergence of new chemotherapy methods, the survival rate of patients with colorectal cancer has improved significantly, but the side effects of these drugs have shown that they are not effective at treating the disease. This has prompted the search for new treatment options. In this context, various types of plants have been shown to have potent apoptogenic and cytotoxic properties [33].

*T. peltata* is rich in catechins such as gallocatechin and epigallocatechin. Studies have shown that green tea, which consists of catechins, can improve metabolic syndrome. (−)-Epigallocatechin-3-gallate has been shown to non-competitively bind to alpha-amylase and can therefore reduce postprandial hyperglycemia [34]. Catechins have potent antioxidant properties and may act as cellular pro-oxidants. These compounds are hence beneficial in protecting the body against a plethora of non-communicable diseases [35]. Catechins are also potent anticancer and chemoprotective molecules working to protect against the effect of carcinomas [36]. Kaempferol is an excellent antioxidant with a very good safety profile. Several in vitro studies have demonstrated the benefits of kaempferol on HepG2 cell lines. In hepatic tumor cells, kaempferol stimulates autophagy via AKT and AMPK signaling molecules and initiates G2/M arrest by causing downregulation of CDK1/cyclin B in SK-HEP-1 in tumor cells of human origin. Kaempferol also acts on carcinoma cells through a variety of other mechanisms [37].

## 4. Materials and Methods

### 4.1. Plant Materials

*Tambourissa peltata* Baker was collected from MonVert nature park in 2019. Samples (fruits, flowers and leaves) of the collected plant species were deposited at the Mauritius Herbarium of the Mauritius Sugarcane Industry and Research Institute (MSIRI) situated in Réduit, Mauritius, for validation of their identity. The identified plant specimen was assigned the barcode number MAU0029104, and a voucher specimen of the species is available at the Mauritius Herbarium.

### 4.2. Extraction of Phytochemicals

The extraction protocols used were as described previously [38,39]. The leaves of the collected plant species were thoroughly washed with distilled water to rid them of debris and left to dry in a well-ventilated area away from direct sunlight exposure. The loss in mass of the leaves was recorded on a daily basis, and after 3 weeks when a constant mass was attained, the dried leaves were pulverized in a mechanical grinder. This process was performed in a beaker by macerating 100 g of each dried plant material separately in 1 L of: (1) ethyl acetate and (2) methanol. The maceration process lasted 14 days, and the beakers were constantly shaken. Each of the mixtures was filtered with grade 1 Whatman^®^ filter paper [38].

A decoction was also prepared by mixing 50 g of dried plant powder in 200 mL of distilled water and heating the resultant mixture at 100 °C for 15 min until the volume decreased to 25% of the original volume [39]. The mixture was then filtered through a muslin cloth. All the filtrates (both organic and aqueous) were concentrated in a rotary evaporator at low temperature and pressure. The resultant crude extracts were preserved at 4 °C in the dark for phytochemical screening and in vitro assays. Before preserving the extracts at 4 °C, they were lyophilized employing a freeze-dryer.

### 4.3. Phytochemical Composition

The bioactive compounds of *T. peltata*, such as its total phenolic, flavonoid, phenolic acid and flavonol contents were determined using colorimetric methods (Folin–Ciocalteu, AlCl_3_, Arnow and DMACA (p-dimethylaminocinnamaldehyde) methods, respectively). The results of these assays were expressed as mg of gallic acid, rutin, caffeic acid and catechin per g of dried extract [40,41]. All the assays were carried out in triplicate. The results are expressed as mean values and standard deviation (SD). The differences between the different extracts were analyzed using one-way analysis of variance (ANOVA) followed by Tukey’s honestly significant difference post hoc test with *p* = 0.05. This treatment was carried out using the SPSS v. 14.0 program (SPSS Inc., Chicago, IL, USA).

### 4.4. HPLC-MS Analysis

Chromatographic analyses were performed with an Agilent Series 1100 with a G1315B diode array detector (Agilent Technologies, Santa Clara, CA, USA), a reversed-phase Luna Omega Polar C_18_ analytical column (150 × 3.0 mm; 5 µm particle size; Phenomenex, Torrance, CA, USA) and a Polar C_18_ Security Guard cartridge of 4 × 3.0 mm (Phenomenex). The mobile phases consisted of water + formic acid 0.1% *v/v* (eluent A) and acetonitrile (eluent B). The gradient elution was: 10–25% B in 0–25 min, 25% B in 25–30 min and 25–100% B in 30–35 min. Then, eluent B was returned to 10% with a 7 min stabilization time. The flow rate was 0.4 mL min^−1^.

The HPLC system was connected to an ion trap mass spectrometer (Esquire 6000, Bruker Daltonics, Billerica, MA, USA) equipped with an electrospray interface. The scan range was at *m/z* 100–1200 with a speed of 13,000 Da/s. The ESI conditions were drying gas (N_2_) flow rate and temperature, 10 L/min and 365 °C; nebulizer gas (N_2_) pressure, 50 psi; capillary voltage, 4500 V; capillary exit voltage, −117.3 V. The auto MS^n^ mode was used for the acquisition of MS^n^ data, with isolation width of 4.0 *m/z* and fragmentation amplitude of 0.6 V.

### 4.5. Evaluation of Biological Activities 

Antioxidant activity (DPPH and ABTS radical scavenging), reducing power (CUPRAC and FRAP), phosphomolybdenum and metal chelating (ferrozine method), enzyme inhibitory activities (cholinesterase (ChE), Elmann’s method), tyrosinase (dopachrome method), α-amylase (iodine/potassium iodide method) and α -glucosidase (chromogenic PNPG method) were determined using the methods previously described by Uysal et al. [42]. Unless stated otherwise, reagents were obtained from Sigma.

For the DPPH (1,1-diphenyl-2-picrylhydrazyl) radical scavenging assay, sample solution (1 mg/mL; 1 mL) was added to 4 mL of a 0.004% methanolic solution of DPPH. The sample absorbance was read at 517 nm after a 30 min incubation at room temperature in the dark. DPPH radical scavenging activity was expressed as millimoles of Trolox equivalents (mg TE/g extract).

For the ABTS (2,2′-azino-bis(3-ethylbenzothiazoline) 6-sulfonic acid) radical scavenging assay, briefly, ABTS^+^ was produced directly by reacting 7 mM ABTS solution with 2.45 mM potassium persulfate and allowing the mixture to stand for 12–16 min in the dark at room temperature. Before beginning the assay, ABTS solution was diluted with methanol and adjusted to an absorbance of 0.700 ± 0.02 at 734 nm. Sample solution (1 mg/mL; 1 mL) was added to ABTS solution (2 mL) and mixed. The sample absorbance was read at 734 nm after a 30 min incubation at room temperature. The ABTS radical scavenging activity was expressed as millimoles of Trolox equivalents (mmol TE/g extract) [43].

For the cupric-ion-reducing activity (CUPRAC) assay, sample solution (1 mg/mL; 0.5 mL) was added to a premixed reaction mixture containing CuCl_2_ (1 mL, 10 mM), neocuproine (1 mL, 7.5 mM) and NH_4_Ac buffer (1 mL, 1 M, pH 7.0). Similarly, a blank was prepared by adding sample solution (0.5 mL) to the premixed reaction mixture (3 mL) without CuCl_2_. Then, the sample and blank absorbances were read at 450 nm after a 30 min incubation at room temperature. The absorbance of the blank was subtracted from that of the sample. CUPRAC activity was expressed as milligrams of Trolox equivalents (mg TE/g extract).

For the ferric-reducing antioxidant power (FRAP) activity assay, sample solution (1 mg/mL; 0.1 mL) was added to premixed FRAP reagent (2 mL) containing acetate buffer (0.3 M, pH 3.6), 2,4,6-tris(2-pyridyl)-*s*-triazine (TPTZ) (10 mM) in 40 mM HCl and ferric chloride (20 mM) in a ratio of 10:1:1 (*v/v/v*). Then, the sample absorbance was read at 593 nm after a 30 min incubation at room temperature. FRAP activity was expressed as milligrams of Trolox equivalents (mg TE/g extract).

For the phosphomolybdenum method, sample solution (1 mg/mL; 0.3 mL) was combined with 3 mL of reagent solution (0.6 M sulfuric acid, 28 mM sodium phosphate and 4 mM ammonium molybdate). The sample absorbance was read at 695 nm after a 90 min incubation at 95 °C. The total antioxidant capacity was expressed as millimoles of Trolox equivalents (mmol TE/g extract) [43]

For the metal chelating activity assay, briefly, sample solution (1 mg/mL; 2 mL) was added to FeCl_2_ solution (0.05 mL, 2 mM). The reaction was initiated by the addition of 5 mM ferrozine (0.2 mL). Similarly, a blank was prepared by adding sample solution (2 mL) to FeCl_2_ solution (0.05 mL, 2 mM) and water (0.2 mL) without ferrozine. Then, the sample and blank absorbances were read at 562 nm after 10 min incubation at room temperature. The absorbance of the blank was subtracted from that of the sample. The metal chelating activity was expressed as milligrams of EDTA (disodium edetate) equivalents (mg EDTAE/g extract).

For the ChE inhibitory activity assay, sample solution (1 mg/mL; 50 μL) was mixed with DTNB (5,5-dithio-bis(2-nitrobenzoic) acid; Sigma, St. Louis, MO, USA) (125 μL) and AChE (acetylcholinesterase (electric ell AChE, Type-VI-S, EC 3.1.1.7, Sigma)) or BChE (BChE (horse serum BChE, EC 3.1.1.8, Sigma)) solution (25 μL) in Tris–HCl buffer (pH 8.0) in a 96-well microplate and incubated for 15 min at 25 °C. The reaction was then initiated with the addition of acetylthiocholine iodide (ATCI, Sigma) or butyrylthiocholine chloride (BTCl, Sigma) (25 μL). Similarly, a blank was prepared by adding sample solution to all reaction reagents without enzyme (AChE or BChE) solution. The sample and blank absorbances were read at 405 nm after 10 min incubation at 25 °C. The absorbance of the blank was subtracted from that of the sample, and the cholinesterase inhibitory activity was expressed as galantamine equivalents (mg GALAE/g extract) [43].

For the tyrosinase inhibitory activity assay, sample solution (1 mg/mL; 25 μL) was mixed with tyrosinase solution (40 μL, Sigma) and phosphate buffer (100 μL, pH 6.8) in a 96-well microplate and incubated for 15 min at 25 °C. The reaction was then initiated with the addition of l-DOPA (40 μL, Sigma). Similarly, a blank was prepared by adding sample solution to all reaction reagents without enzyme (tyrosinase) solution. The sample and blank absorbances were read at 492 nm after a 10 min incubation at 25 °C. The absorbance of the blank was subtracted from that of the sample, and the tyrosinase inhibitory activity was expressed as kojic acid equivalents (mg KAE/g extract) [43].

For the α-amylase inhibitory activity assay, sample solution (1 mg/mL; 25 μL) was mixed with α-amylase solution (ex-porcine pancreas, EC 3.2.1.1, Sigma) (50 μL) in phosphate buffer (pH 6.9 with 6 mM sodium chloride) in a 96-well microplate and incubated for 10 min at 37 °C. After pre-incubation, the reaction was initiated with the addition of starch solution (50 μL, 0.05%). Similarly, a blank was prepared by adding sample solution to all reaction reagents without enzyme (α-amylase) solution. The reaction mixture was incubated for 10 min at 37 °C. The reaction was then stopped with the addition of HCl (25 μL, 1 M). This was followed by the addition of the iodine–potassium iodide solution (100 μL). The sample and blank absorbances were read at 630 nm. The absorbance of the blank was subtracted from that of the sample, and the α-amylase inhibitory activity was expressed as acarbose equivalents (mmol ACE/g extract) [44].

For the α-glucosidase inhibitory activity assay, sample solution (1 mg/mL; 50 μL) was mixed with glutathione (50 μL), α-glucosidase solution (from *Saccharomyces cerevisiae*, EC 3.2.1.20, Sigma) (50 μL) in phosphate buffer (pH 6.8) and PNPG (4-nitrophenyl-β-d-glucopyranoside, Sigma) (50 μL) in a 96-well microplate and incubated for 15 min at 37 °C. Similarly, a blank was prepared by adding sample solution to all reaction reagents without enzyme (α-glucosidase) solution. The reaction was then stopped with the addition of sodium carbonate (50 μL, 0.2 M). The sample and blank absorbances were read at 400 nm. The absorbance of the blank was subtracted from that of the sample, and the α-glucosidase inhibitory activity was expressed as acarbose equivalents (mmol ACE/g extract) [45].

All the assays were carried out in triplicate. The results are expressed as mean values and standard deviation (SD). The differences between the different extracts were analyzed using one-way analysis of variance (ANOVA) followed by Tukey’s honestly significant difference post hoc test with *p* = 0.05. This treatment was carried out using SPSS v. 14.0 program.

### 4.6. Cell Viability Assay

The cell viability assay was conducted to analyze the toxicity level of aqueous and methanolic extract to cell lines, which were normal human fibroblasts (3T3), human hepatocellular carcinoma cells (HepG2) and human colorectal carcinoma cells (HT29), purchased from ATCC (Manassas, VA, USA). All the cells were grown using Roswell Park Memorial Institute (RPMI) 1640 medium (Nacalai Tesque, Kyoto, Japan) supplemented with 10% fetal bovine albumin (Sigma-Aldrich, St. Louis, MO, USA), 1% antibiotics containing 10,000 units/mL penicillin and 10,000 μg/mL streptomycin (Nacalai Tesque, Kyoto, Japan). Cells were maintained and incubated in humidified 5% carbon dioxide, 95% room air, at 37 °C. Cells layers were harvested using 0.25% trypsin/1 mM-EDTA (Nacalai Tesque, Kyoto, Japan). This was followed by seeding in 96-well tissue culture plates at 1.0 × 10^4^ cells/well in an incubator to attach and attain 80% confluence for treatment after 24 h.

The methylthiazol tetrazolium (MTT)-based assay was carried out to determine the cell viability and cytotoxicity. Cells were treated with *T. peltata* in aqueous and methanolic extracts, where stock solutions were prepared by dissolving the compound in 1:1 of dimethyl sulfoxide (0.1%) and Roswell Park Memorial Institute (RPMI) medium. Then, the mixture was further diluted in the same media to produce various final concentrations, ranging from 31.25 to 1000 μg/mL.

Once the cells were attached to the respective wells after 24 h, the tested compounds were added until the final volume of 100 μL well was obtained. After 48 h of incubation, 10 μL of MTT solution (5 mg/mL in PBS) was added to each well and further incubated for 3 h before being aspirated. Then, 100 μL of dimethyl sulfoxide was added per well in the dark and room temperature in order to dissolve the purple formazan salt. The intensity of the purple formazan solution, which reflects cell growth, was subsequently measured at a wavelength of 570 nm using a microplate reader (Biotek LE800, Winooski, VT, USA). All experiments were carried out in triplicate, and the results are presented as the mean ± standard deviation. IC_50_ was measured when required.

All the cytotoxicity assays were carried out in triplicate, and the standard deviations were calculated and incorporated in the respective bar graphs. For the calculation of IC_50_, we plotted the *x*-axis against the *y*-axis and converted the *x*-axis values (concentration) to their log values, followed by nonlinear regression (curve fit) in the xy analysis to obtain a straight-line equation fit, y = ax + b, from which the regression line and then inhibition IC_50_ were calculated.

### 4.7. Cytotoxicity Studies on Normal (NIH 3T3) and Cancer (HepG2 and HT 29) Cell Lines

Cytotoxicity studies were conducted by treating with *T. peltata* in aqueous, methanolic and ethyl acetate extracts on normal fibroblast, 3T3 and cancer (HepG2 and HT 29) cell lines. Various gradient concentrations of the samples were incubated for a maximum of 48 h.

## 5. Conclusions

This work has enabled for the first time the study of *T. peltata* phytochemical constituents and biological properties in vitro. This work can be considered as a stepping stone for further study of this plant species. Based on the results, it can be concluded that different sample treatments can impact bioactivities. Hence, we recommend that the same proportion of plant and solvent are used for each extraction, and future studies will be designed to compare such parameters. Given its exceptional antioxidant properties, which were higher than the reference compound used, coupled with its richness in phytoconstituents, which have notable pharmacological properties, this plant species undeniably deserves more attention from the scientific community. Its activity against acetylcholinesterases, α-amylase and glucosidase as well as tyrosinase enzymes underscores the biological potential of this plant species. Given the excellent antioxidant properties of *T. peltata*, a plethora of potential applications can be derived from this plant species: (i) it can be prepared and marketed as a dietary supplement pending further toxicological studies on this species, and (ii) it can be used as a complementary therapy in the management of non-communicable diseases such as dementia, diabetes mellitus and skin conditions, following the rigorous herb–drug interaction that it may or may not induce when combined with other therapies. Its potential against colorectal carcinoma also cannot be ignored, and given its endemic nature, word must be spread for its conservation in an attempt to ensure its sustainable use.

## Figures and Tables

**Table 1:** Phytochemical profile of the extracts.

Extract	Total Phenolic Content (mg GAE/g)	Total Flavonoid Content (mg RE/g)	Total Phenolic Acid Content (mg CAE/g)	Total Flavonol Content (mg CE/g)
TP-Aq	179.91 ± 0.67 ^a^	19.94 ± 0.34 ^c^	55.74 ± 1.43 ^a^	3.60 ± 0.08 ^c^
TP-EA	64.51 ± 0.56 ^c^	28.97 ± 0.46 ^a^	0	19.63 ± 0.42 ^b^
TP-MEOH	129.79 ± 0.72 ^b^	24.85 ± 0.45 ^b^	9.32 ± 1.45 ^b^	33.71 ± 0.13 ^a^

TP-Aq: *Tambourissa peltata* Baker aqueous extract; TP-EA: *T. peltata* ethylacetate extract; TP-MEOH: *T. peltata* methanolic extract; GAE: gallic acid equivalent; RE: rutin equivalent; CAE: caffeic acid equivalent; CE: catechin equivalent. Different letters (a–c) indicate significant differences between the tested extracts (*p* < 0.05).

**Table 2:** Characterization of the compounds found in the analyzed extracts of *Tambourissa peltata* Baker.

No.	t*_R_* (min)	[M − H]^−^ *m/z*	*m/z* (% Base Peak)	H_2_O	EA	MeOH	Assigned Identification
1	1.7	377	MS^2^ [377]: 341 (100), 179 (15) MS^3^ [377→341]: 179 (100), 143 (11), 131 (11) MS^4^ [377→341→179]: 161 (78), 149 (65), 119 (100), 113 (53)	✓		✓	Disaccharide (HCl adduct)
2	2.1	191	MS^2^ [191]: 173 (48), 111 (100)	✓			Isocitric acid
3	2.7	609	MS^2^ [609]: 591 (27), 483 (22), 471 (17), 441 (84), 423 (100), 305 (38)	✓	✓	✓	Prodelphinidin dimer B (two units of (epi)GC)
4	3.1	865	MS^2^ [865]: 739 (12), 695 (100), 577 (18), 575 (31), 451 (16), 407 (22), 287 (19) MS^3^ [865→695]: 587 (35), 543 (100), 525 (37), 289 (32), 243 (59)	✓			Procyanidin trimer B-type
5	3.5	609	MS^2^ [609]: 591 (7), 483 (15), 441 (100), 423 (89), 305 (41)	✓	✓	✓	Prodelphinidin dimer B (two units of (epi)GC)
6	3.6	593	MS^2^ [593]: 467 (25), 441 (27), 425 (100), 423 (47), 407 (43), 305 (26), 289 (24)	✓	✓	✓	Prodelphinidin dimer B-type (one unit of (epi)GC)
7	4.3	593	MS^2^ [593]: 467 (53), 441 (79), 425 (35), 423 (100), 305 (74), 287 (8)	✓			Prodelphinidin dimer B-type (one unit of (epi)GC)
8	4.5	305	MS^2^ [305]: 261 (20), 221 (52), 219 (69), 179 (100), 165 (30), 125 (31)	✓	✓	✓	Gallocatechin
9	5.3	365	MS^2^ [365]: 321 (55), 211 (65), 153 (100) MS^3^ [365→153]: 109 (100)	✓		✓	Dihydroxybenzoic acid derivative
10	5.6	593	MS^2^ [593]: 467 (11), 425 (100), 407 (57), 289 (20)	✓	✓	✓	Prodelphinidin dimer B-type (one unit of (epi)GC)
11	5.7	761	MS^2^ [761]: 609 (98), 591 (54), 465 (22), 441 (52), 423 (100), 305 (34) MS^3^ [761→609]: 591 (32), 483 (85), 423 (40), 305 (100), 177 (36)	✓		✓	Prodelphinidin dimer B-type gallate (two units (epi)GC)
12	6.8	577	MS^2^ [577]: 451 (28), 425 (100), 407 (68), 289 (24), 287 (20), 245 (11)	✓	✓		Procyanidin dimer B-type
13	7.1	305	MS^2^ [305]: 261 (21), 221 (45), 219 (75), 179 (100), 165 (27), 125 (42)	✓	✓	✓	Epigallocatechin
14	7.5	593	MS^2^ [593]: 575 (30), 467 (31), 441 (84), 425 (100), 423 (92), 305 (52), 289 (28)	✓	✓	✓	Prodelphinidin dimer B-type (one unit of (epi)GC)
15	8.0	761	MS^2^ [761]: 743 (30), 635 (38), 609 (65), 593 (72), 591 (43), 575 (70), 457 (30), 423 (100) MS^3^ [761→593]: 575 (26), 405 (19), 423 (100)	✓		✓	Prodelphinidin dimer B-type gallate (two units (epi)GC)
16	8.9	289	MS^2^ [289]: 245 (100), 205 (34), 203 (15), 179 (11), 151 (9)	✓	✓	✓	Catechin
17	10.6	431	MS^2^ [431]: 385 (100), 223 (10), 153 (9) MS^3^ [431→385]: 223 (100), 205 (39), 161 (52), 153 (73), 138 (9)	✓	✓	✓	Roseoside (formate adduct)
18	10.8	577	MS^2^ [577]: 451 (10), 425 (100), 407 (95), 289 (26), 287 (8)	✓	✓	✓	Procyanidin dimer B-type
19	12.0	289	MS^2^ [289]: 245 (100), 205 (33), 203 (15), 179 (13)	✓	✓	✓	Epicatechin
20	14.2	865	MS^2^ [865]: 713 (33), 695 (100), 577 (45), 575 (36), 451 (33), 425 (30), 407 (53), 289 (41)	✓	✓	✓	Procyanidin trimer B-type
21	14.3	593	MS^2^ [593]: 575 (14), 467 (23), 441 (30), 425 (100), 423 (58), 407 (51), 305 (27)	✓	✓	✓	Prodelphinidin dimer B-type (one unit of (epi)GC)
22	16.7	479	MS^2^ [479]: 317 (79), 316 (100), 179 (9) MS^3^ [479→316]: 271 (100), 179 (40), 151 (17)	✓	✓	✓	Myricetin-*O*-hexoside
23	17.1	479	MS^2^ [479]: 317 (99), 316 (100), 179 (12) MS^3^ [479→316]: 271 (79), 179 (100), 151 (27)	✓		✓	Myricetin-*O*-hexoside
24	18.0	449	MS^2^ [449]: 317 (100), 316 (94) MS^3^ [449→317]: 271 (100), 179 (86), 151 (41)	✓	✓	✓	Myricetin-*O*-pentoside
25	19.3	449	MS^2^ [449]: 317 (40), 316 (100) MS^3^ [449→316]: 271 (100), 179 (13), 151 (22)	✓		✓	Myricetin-*O*-pentoside
26	19.8	463	MS^2^ [463]: 317 (70), 316 (100) MS^3^ [463→316]: 217 (100), 179 (29), 151 (17)	✓	✓	✓	Myricetin-*O*-deoxyhexoside
27	20.5	463	MS^2^ [463]: 301 (100) MS^3^ [463→301]: 179 (100), 151 (88)	✓	✓	✓	Quercetin-*O*-hexoside
28	22.3	415	MS^2^ [415]: 369 (21), 225 (21), 179 (100), 161 (10)	✓	✓	✓	Unknown
29	23.2	433	MS^2^ [433]: 301 (100) MS^3^ [433→301]: 271 (56), 255 (100), 179 (75), 151 (33)	✓	✓	✓	Quercetin-*O*-pentoside
30	24.4	477	MS^2^ [477]: 331 (100), 316 (34) MS^3^ [477→331]: 316 (100), 271 (2) MS^4^ [477→331→316]: 316 (100), 287 (30), 271 (50), 179 (43), 136 (60)	✓	✓	✓	Mearnsetin-*O*-deoxyhexoside
31	24.7	447	MS^2^ [447]: 301 (100) MS^3^ [447→301]: 179 (77), 151 (100)	✓	✓	✓	Quercetin-*O*-deoxyhexoside
32	26.8	317	MS^2^ [317]: 271 (5), 179 (100), 151 (46)		✓	✓	Myricetin
33	28.7	431	MS^2^ [431]: 285 (100), 255 (8) MS^3^ [431→285]: 257 (55), 255 (100), 227 (24), 241 (17)	✓	✓	✓	Kaempferol-*O*-deoxyhexoside
34	29.4	461	MS^2^ [461]: 315 (100) MS^3^ [461→315]: 300 (100), 271 (6)	✓	✓	✓	Isorhamnetin-*O*-deoxyhexoside

**Table 3:** Quantification of the main phytochemicals in the analyzed extract of *T. peltata* (mg g^−1^ dry extract (DE); *n* = 3).

No.	Assigned Identification	H_2_O	EA	MeOH
Proanthocyanidins				
3	Prodelphinidin dimer	14.1 ± 0.7	1.13 ± 0.08	--
4	Procyanidin trimer	2.4 ± 0.2	--	--
5 + 6	Prodelphinidin dimers	3.2 ± 0.2	--	2.2 ± 0.1
10 + 11	Prodelphinidin dimers	3.0 ± 0.2	0.79 ± 0.05	0.78 ± 0.05
12	Procyanidin dimer	2.2 ± 0.1	0.17 ± 0.01	--
14	Prodelphinidin dimer	1.4 ± 0.07	0.24 ± 0.02	0.36 ± 0.02
20 + 21	Procyanidin trimer + prodelphinidin dimer	1.38 ± 0.08	--	0.59 ± 0.03
**Total**		**27.7 ± 0.8**	**2.3 ± 0.1**	**3.9 ± 0.1**
Flavonoids				
8	Gallocatechin	4.4 ± 0.2	0.133 ± 0.009	--
13	Epigallocatechin	4.3 ± 0.3	0.21 ± 0.01	1.17 ± 0.06
16	Catechin	0.066 ± 0.004	1.5 ± 0.1	2.2 ± 0.1
19	Epicatechin	7.7 ± 0.3	0.72 ± 0.05	4.1 ± 0.2
22	Myricetin-*O*-hexoside	2.1 ± 0.1	0.69 ± 0.04	2.5 ± 0.1
23	Myricetin-*O*-hexoside	0.47 ± 0.03	--	0.28 ± 0.02
24	Myricetin-*O*-pentoside	0.135 ± 0.008	--	--
25	Myricetin-*O*-pentoside	0.121 ± 0.008	--	--
26	Myricetin-*O*-deoxyhexoside	13.4 ± 0.6	16.2 ± 0.8	12.2 ± 0.6
27	Quercetin-*O*-hexoside	0.21 ± 0.01	0.24 ± 0.02	0.27 ± 0.02
29	Quercetin-*O*-pentoside	0.0073 ± 0.0005	0.043 ± 0.003	0.023 ± 0.002
30 + 31	Mearnsetin + quercetin glycosides	3.8 ± 0.2	13.5 ± 0.8	4.4 ± 0.3
32	Myricetin	--	0.55 ± 0.03	--
33	Kaempferol-*O*-deoxyhexoside	0.137 ± 0.008	0.24 ± 0.02	0.130 ± 0.008
34	Isorhamnetin-*O*-deoxyhexoside	0.140 ± 0.008	0.41 ± 0.03	0.15 ± 0.01
**Total**		**37.0 ± 0.8**	**34 ± 1**	**27.4 ± 0.8**
Others				
9	Dihydroxybenzoic acid derivative	0.29 ± 0.02	--	--
**TIPC**		**65 ± 1**	**36 ± 1**	**31.3 ± 0.8**

EA: ethyl acetate; MeOH: methanol; TIPC: total individual phenolic content.

**Table 4:** Antioxidant potential of the assayed extracts.

Samples	DPPH (mg TE/g)	ABTS (mg TE/g)	FRAP (mg TE/g)	CUPRAC (mg TE/g)	Metal Chelating (mg EDTAE/g)	Phosphomolybdenum (mmol TE/g)
TP-Aq	973.40 ± 5.65 ^a^	2030.37 ± 40.83 ^a^	1461.39 ± 5.95 ^a^	1940.99 ± 20.95 ^a^	4.75 ± 0.70 ^a^	8.37 ± 0.23 ^a^
TP-EA	186.80 ± 0.18 ^c^	482.13 ± 0.96 ^c^	357.69 ± 7.77 ^c^	548.62 ± 5.16 ^d^	4.88 ± 0.32 ^a^	3.64 ± 0.26 ^c^
TP-MEOH	399.47 ± 0.73 ^b^	973.23 ± 2.25 ^b^	835.27 ± 23.49 ^b^	1161.15 ± 10.47 ^c^	2.12 ± 0.26 ^b^	5.91 ± 0.41 ^b^

Values are reported as mean ± S.D. of three parallel experiments. Aq: *Tambourissa peltata* Baker aqueous extract; TP-EA: *T. peltata* ethylacetate extract; TP-MEOH: *T. peltata* methanolic extract; TE: Trolox equivalent; EDTAE: EDTA equivalent. Different letters (a–d) indicate significant differences between the tested extracts (*p* < 0.05).

**Table 5:** Enzyme inhibitory potential of the extracts.

Samples	AChE Inhibition (mg GALAE/g)	BChE Inhibition (mg GALAE/g)	Tyrosinase Inhibition (mg KAE/g)	Amylase Inhibition (mmol ACAE/g)	Glucosidase Inhibition (mmol ACAE/g)
TP-Aq	3.49 ± 0.06 ^c^	2.83 ± 0.42 ^c^	124.27 ± 1.77 ^c^	1.37 ± 0.04 ^a^	1.82 ± 0.01 ^a^
TP-EA	3.98 ± 0.10 ^b^	9.76 ± 0.14 ^a^	132.77 ± 0.90 ^b^	0.72 ± 0.07 ^c^	1.70 ± 0.03 ^b^
TP-MEOH	4.87 ± 0.04 ^a^	5.80 ± 0.12 ^b^	155.30 ± 0.55 ^a^	0.92 ± 0.02 ^b^	1.80 ± 0.01 ^a^

Values are reported as mean ± S.D. of three parallel experiments. AChE: acetylcholinesterase; BChE: butyrylcholinesterase; GALAE: galantamine equivalent; KAE: kojic acid equivalent; ACAE: acarbose equivalent. Different letters (a–d) indicate significant differences between the tested extracts (*p* < 0.05).

## Data Availability

Not applicable.
